# Supplementary material for: Genomic and Transcriptional Profiles of Kelch-like (klhl) Gene Family in Polyploid Carassius Complex
Source: Int J Mol Sci. 2023 May 6;24(9):8367. doi: 10.3390/ijms24098367 (PMC10179623; doi:10.3390/ijms24098367)
Supplement: Supplementary file 1 [file ijms-24-08367-s001.zip › ijms-2130123-supplementary.pdf]

Supplementary information

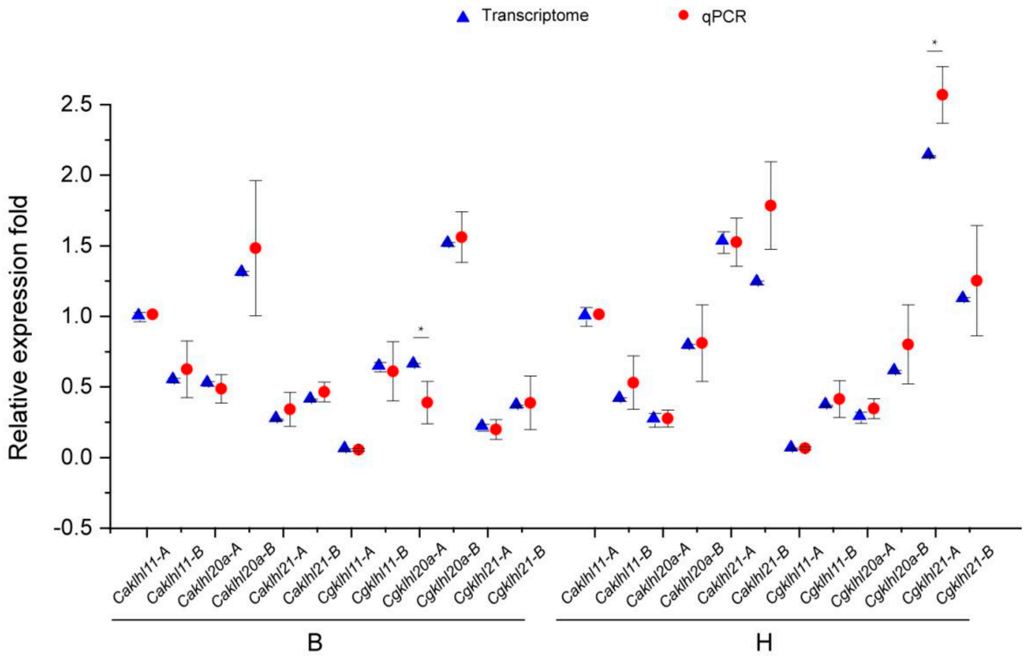

Fig. S1 Gene expression comparisons between the results of transcriptome and those of qPCR. The horizontal axis represents the randomly-selected *C. gibelio* and *C. auratus klhls*, with B and H representing Brain and Heart, respectively. The vertical axis is the normalized gene expression levels based on the value of *Caklhl11-A* in Brain and Heart, respectively. Data are presented by mean ± sd. No significance is observed except *Cgklhl20a-B* in Brain and *Cgklhl21-A* in Heart (* $P < 0.05$, student's t-test).

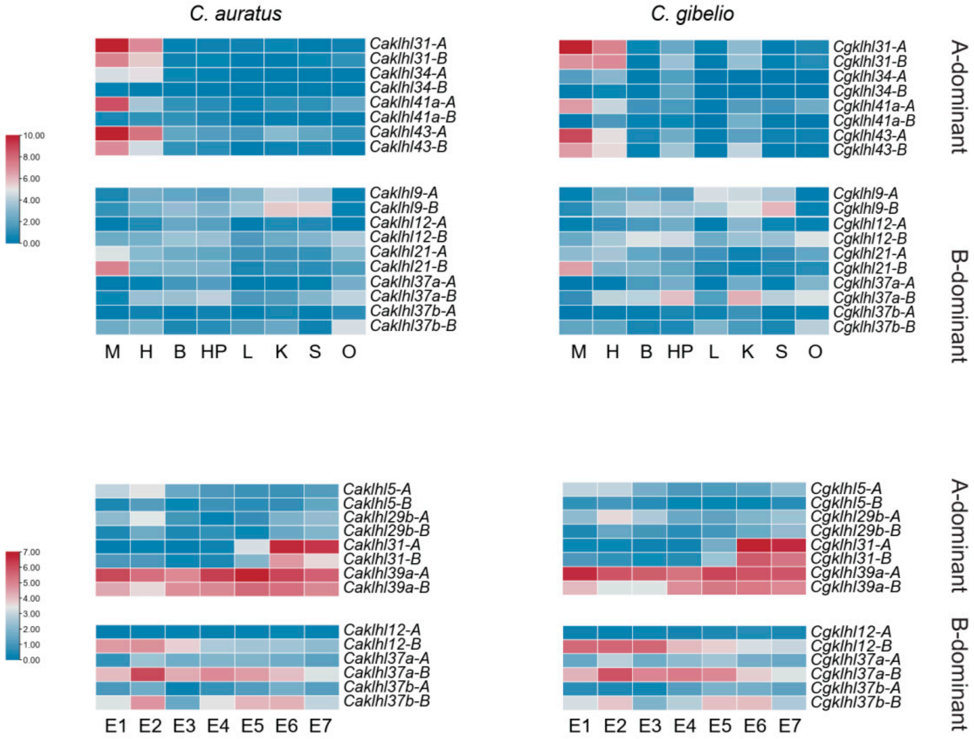

Fig. S2 Heatmaps of common homeolog-dominant genes of *C. auratus* (*Ca*) and *C. gibelio* (*Cg*) in combined analysis. M, Muscle; H, Heart; B, Brain; HP; Hypothalamus-pituitary; L, Liver; K, Kidney; S, Spleen; O, Ovary; E1, 4-cell stage; E2, Blastula stage; E3, Shield stage; E4, Bud stage; E5, 8-somite stage; E6, Prime-5 stage; E7, Pec-fin stage.

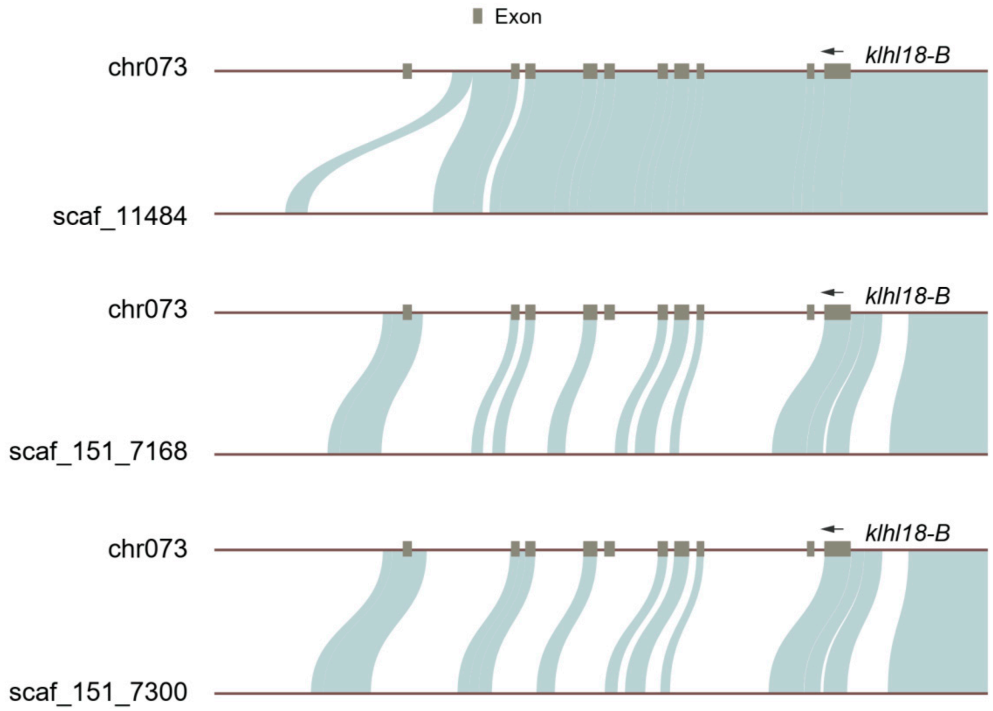

Fig. S3 Sequence comparisons among *Cgklhl18-B* homologous regions in the assembly with 150 chromosome. *Cgklhl18-B* has one allele at chr073 although the assembly has three other homologous regions.

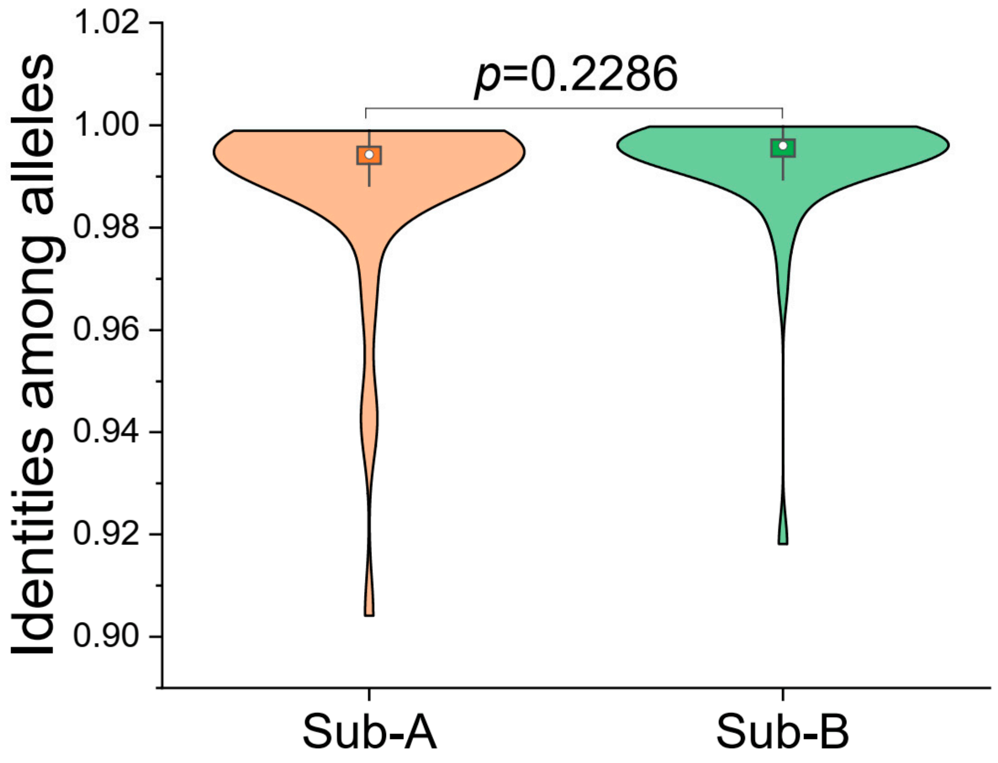

Fig. S4 Violin plot of identities among alleles for *Cgklhl* genes in subgenomes A and B. The circle in the middle of each boxplot represents the median of the dataset.

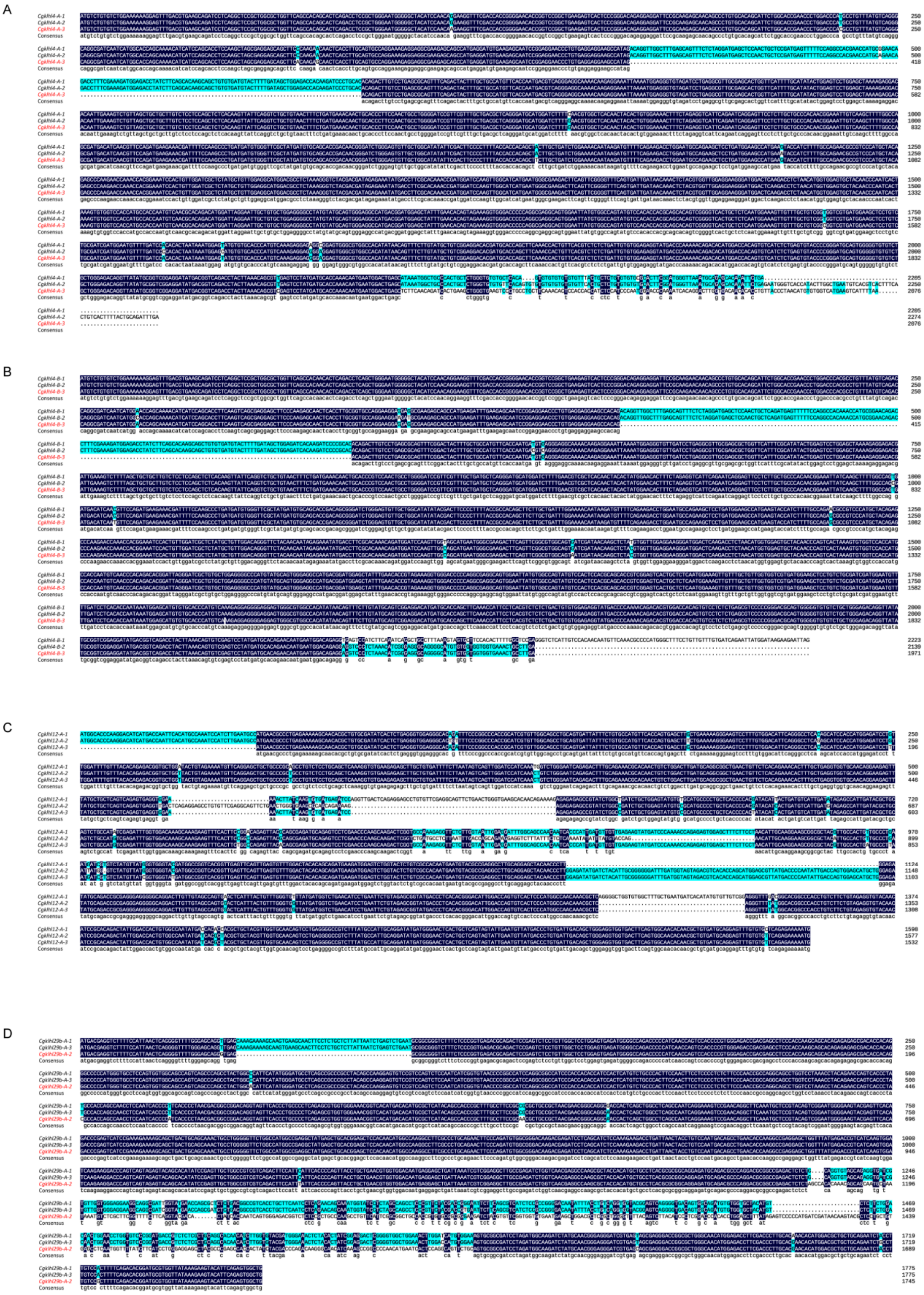

Fig. S5 Sequence alignment of the alleles with identity <90% for four *Cgklhl* genes: *klhl4-A* (A), *klhl4-B* (B), *klhl12-A* (C), *and klhl29b-A* (D). Red allele shows low identities with other alleles because of the fragment deletion.

Table S1 *klhl* genes in 13 animals.

Pefromyzon marinus	***Callorhinclus milii***	***Latimeria clalumnae***	***Gallus gallu***	***Homo sapiens***	***Lepisosteus oculatus***	***Fugu rubripcm***	***Salmo salar***	***Oncorhynclus mykiss***	***Danio rerio***	***Carassius auratus***	***Carsasius gibelio***
	Cmklhl1	*Lcklhl1*	*Clklhl1*	*Hsklhl1*	*Loklhl1*						
Pmklhl2	*Cmklhl2*	*Lcklhl2*	*Clklhl2*	*Hsklhl2*	*Loklhl2*	*Frklhl2*			*Drklhl2.1*<br>*Drklhl2.2*<br>*Drklhl2.3*	*Caklhl2-A*<br>*Caklhl2-B*	*Cgklhl2-A*<br>*Cgklhl2-B*
	Cmklhl3	*Lcklhl3*	*Clklhl3*	*Hsklhl3*	*Loklhl3*	*Frklhl3*	*Saklhl3.1*<br>*Saklhl3.2*	*Omklhl3.1*<br>*Omklhl3.2*	*Drklhl3.1*<br>*Drklhl3.2*	*Caklhl3-A*	*Cgklhl3-A*
	Cmklhl4	*Lcklhl4*	*Clklhl4*	*Hsklhl4*	*Loklhl4*	*Frklhl4*	*Saklhl4.1*<br>*Saklhl4.2*	*Omklhl4.1*<br>*Omklhl4.2*	*Drklhl4*	*Caklhl4-A*<br>*Caklhl4-B*	*Cgklhl4-A*<br>*Cgklhl4-B*
Pmklhl5	*Cmklhl5*	*Lcklhl5*	*Clklhl5*	*Hsklhl5*	*Loklhl5*	*Frklhl5*	*Saklhl5*		*Drklhl5.1*<br>*Drklhl5.2*<br>*Drklhl5.3*	*Caklhl5-A*<br>*Caklhl5-B*	*Cgklhl5-A*<br>*Cgklhl5-B*
		Lcklhl6	*Clklhl6*	*Hsklhl6*	*Loklhl6*	*Frklhl6*	*Saklhl6*	*Omklhl6*	*Drklhl6*	*Caklhl6-A*<br>*Caklhl6-B*	*Cgklhl6-A*<br>*Cgklhl6-B*
	Cmklhl7	*Lcklhl7*	*Clklhl7*	*Hsklhl7*	*Loklhl7*	*Frklhl7*	*Saklhl7*	*Omklhl7*	*Drklhl7*	*Caklhl7-B*	*Cgklhl7-B*
Pmklhl8	*Cmklhl8*		*Clklhl8*	*Hsklhl8*	*Loklhl8*	*Frklhl8*		*Omklhl8.1*<br>*Omklhl8.2*	*Drklhl8*	*Caklhl8-1*<br>*Caklhl8-2*	*Cgklhl8-1*<br>*Cgklhl8-2*<br>*Cgklhl8-A*<br>*Cgklhl8-B*
	Cmklhl9		*Clklhl9.1*<br>*Clklhl9.2*	*Hsklhl9*	*Loklhl9*	*Frklhl9*	*Saklhl9*	*Omklhl9.1*<br>*Omklhl9.2*	*Drklhl9.1*<br>*Drklhl9.2*	*Caklhl9-A*<br>*Caklhl9-B*	*Cgklhl9-A*<br>*Cgklhl9-B*
Pmklhl10	*Cmklhl10*	*Lcklhl10*	*Clklhl10*	*Hsklhl10*	*Loklhl10*	*Frklhl10a*			*Drklhl10a*	*Caklhl10a-B*	*Cgklhl10a-B*
						Frklhl10b	*Saklhl10b.1*	*Omklhl10b.1*	*Drklhl10b.1*	*Caklhl10b-A*	*Cgklhl10b-A*

							Saklhl10b.2	*Omklhl10b.2*	*Drklhl10b.2*	*Caklhl10b-B*	*Cgklhl10b-B*
							Saklhl10b.3	*Omklhl10b.3*	*Drklhl10b.3*		
	Cmklhl11	*Lcklhl11*	*Clklhl11*	*Hsklhl11*	*Loklhl11*	*Frklhl11*	*Saklhl11.1*	*Omklhl11.1*	*Drklhl11*	*Caklhl11-A*	*Cgklhl11-A*
							Saklhl11.2	*Omklhl11.2*		*Caklhl11-B*	*Cgklhl11-B*
							Saklhl11.3	*Omklhl11.3*			
Pmklhl12	*Cmklhl12*	*Lcklhl12*	*Clklhl12*	*Hsklhl12*	*Loklhl12*	*Frklhl12*	*Saklhl12*	*Omklhl12*	*Drklhl12.1*	*Caklhl12-A*	*Cgklhl12-A*
									Drklhl12.2	*Caklhl12-B*	*Cgklhl12-B*
Pmklhl13	*Cmklhl13*	*Lcklhl13.1*		*Hsklhl13*	*Loklhl13*	*Frklhl13*	*Saklhl13.1*		*Drklhl13.1*	*Caklhl13-A*	*Cgklhl13-A*
		Lcklhl13.2					*Saklhl13.2*		*Drklhl13.2*	*Caklhl13-B*	*Cgklhl13-B*
							Saklhl13.3		*Drklhl13.3*		
									Drklhl13-like	*Caklhl13-like*	*Cgklhl13-like*
	Cmklhl14	*Lcklhl14*	*Clklhl14*	*Hsklhl14*	*Loklhl14*	*Frklhl14*	*Saklhl14.1*	*Omklhl14.1*	*Drklhl14*	*Caklhl14-A*	*Cgklhl14-A*
							Saklhl14.2	*Omklhl14.2*		*Caklhl14-B*	*Cgklhl14-B*
	Cmklhl15	*Lcklhl15*	*Clklhl15*	*Hsklhl15*	*Loklhl15*	*Frklhl15*	*Saklhl15.1*	*Omklhl15.1*	*Drklhl15*	*Caklhl15-A*	*Cgklhl15-A*
							Saklhl15.2	*Omklhl15.2*		*Caklhl15-B*	*Cgklhl15-B*
Pmklhl16	*Cmklhl16*	*Lcklhl16*	*Clklhl16*	*Hsklhl16*	*Loklhl16*	*Frklhl16*	*Saklhl16.1*	*Omklhl16.1*	*Drklhl16*	*Caklhl16-B*	*Cgklhl16-B*
							Saklhl16.2	*Omklhl16.2*			
	Cmklhl17	*Lcklhl17*	*Clklhl17*	*Hsklhl17*	*Loklhl17*	*Frklhl17a*			*Drklhl17a*	*Caklhl17a-A*	*Cgklhl17a-A*
										Caklhl17a-B	*Cgklhl17a-B*
						Frklhl17b	*Saklhl17b.1*	*Omklhl17b*	*Drklhl17b.1*	*Caklhl17b-A*	*Cgklhl17b-B1*
							Saklhl17b.2	*Omklhl17b.1*	*Drklhl17b.2*	*Caklhl17b-B*	*Cgklhl17b-B2*
Pmklhl18		*Lcklhl18*	*Clklhl18*	*Hsklhl18*	*Loklhl18*	*Frklhl18*	*Saklhl18*	*Omklhl18*	*Drklhl18*	*Caklhl18-A*	*Cgklhl18-A*
										Caklhl18-B	*Cgklhl18-B*
	Cmkeap1a	*Lckeap1a*		*Hskeap1*	*Lokeap1a*	*Frkeap1a*	*Sakeap1a.1*	*Omkeap1a*	*Drkeap1a*	*Cakeap1a-A*	*Cgkeap1a-A*
							Sakeap1a.2			*Cakeap1a-B*	*Cgkeap1a-B*
	Cmkeap1b	*Lckeap1b*			*Lokeap1b*	*Frkeap1b*	*Sakeap1b.1*	*Omkeap1b*	*Drkeap1b*	*Cakeap1b-A*	*Cgkeap1b-B*

							Sakeap1b.2				
Pmklhl20	*Cmklhl20*	*Lcklhl20*	*Clklhl20*	*Hsklhl20*	*Loklhl20*	*Frklhl20a*			*Drklhl20a*	*Caklhl20a-A*	*Cgklhl20a-A*
										Caklhl20a-B	*Cgklhl20a-B*
						Frklhl20b	*Saklhl20b.1*	*Omklhl20b.1*	*Drklhl20b*	*Caklhl20b-A*	*Cgklhl20b-A*
							Saklhl20b.2	*Omklhl20b.2*		*Caklhl20b-B*	*Cgklhl20b-B*
Pmklhl21	*Cmklhl21*	*Lcklhl21*	*Clklhl21*	*Hsklhl21*	*Loklhl21*	*Frklhl21*	*Saklhl21.1*	*Omklhl21*	*Drklhl21*	*Caklhl21-A*	*Cgklhl21-A*
							Saklhl21.2			*Caklhl21-B*	*Cgklhl21-B*
Pmklhl22	*Cmklhl22*	*Lcklhl22*	*Clklhl23*	*Hsklhl22*	*Loklhl22*	*Frklhl22*	*Saklhl22.1*	*Omklhl22*	*Drklhl22*	*Caklhl22-B*	*Cgklhl22-B*
							Saklhl22.2				
Pmklhl23	*Cmklhl23*	*Lcklhl23*	*Clklhl24*	*Hsklhl23*	*Loklhl23*	*Frklhl23*	*Saklhl23.1*	*Omklhl23.1*	*Drklhl23.1*	*Caklhl23.1-A*	*Cgklhl23.1-A*
							Saklhl23.2	*Omklhl23.2*	*Drklhl23.2*	*Caklhl23.2-A*	*Cgklhl23.2-A*
Pmklhl24	*Cmklhl24*	*Lcklhl24*		*Hsklhl24*	*Loklhl24*	*Frklhl24*			*Drklhl24a*	*Caklhl24a-A*	*Cgklhl24a-A*
										Caklhl24a-B	*Cgklhl24a-B*
							Saklhl24b.1	*Omklhl24b.1*	*Drklhl24b*	*Caklhl24b-A*	*Cgklhl24b-A*
							Saklhl24b	*Omklhl24b.2*		*Caklhl24b-B*	*Cgklhl24b-B*
		Lcklhl25	*Clklhl25*	*Hsklhl25*	*Loklhl25*	*Frklhl25*			*Drklhl25*	*Caklhl25-A1*	*Cgklhl25-A*
										Caklhl25-A2	*Cgklhl25-B*
Pmklhl26	*Cmklhl26*	*Lcklhl26*		*Hsklhl26*	*Loklhl26*	*Frklhl26*	*Saklhl26.1*	*Omklhl26.1*	*Drklhl26*	*Caklhl26-A*	*Cgklhl26-A*
							Saklhl26	*Omklhl26.2*		*Caklhl26-B*	*Cgklhl26-B*
	Cmklhl27	*Lcklhl27*	*Clklhl27*	*Hsklhl27*	*Loklhl27*	*Frklhl27*	*Saklhl27*	*Omklhl27*		*Caklhl27-B*	*Cgklhl27-B*
	Cmklhl28	*Lcklhl28*	*Clklhl28*	*Hsklhl28*	*Loklhl28*	*Frklhl28*	*Saklhl28.1*	*Omklhl28.1*	*Drklhl28*	*Caklhl28-A*	*Cgklhl28-A*
							Saklhl28.2	*Omklhl28.2*		*Caklhl28-B*	*Cgklhl28-B*
Pmklhl29	*Cmklhl29*	*Lcklhl29*		*Hsklhl29*	*Loklhl29*	*Frklhl29*			*Drklhl29a*	*Caklhl29a-A*	*Cgklhl29a-A*
										Caklhl29a-B	*Cgklhl29a-B*
							Saklhl29b.1	*Omklhl29b.1*	*Drklhl29b*	*Caklhl29b-A*	*Cgklhl29b-A*
							Saklhl29b.2	*Omklhl29b.2*		*Caklhl29b-B*	*Cgklhl29b-B*

	Cmklhl30	*Lcklhl30*	*Clklhl30*	*Hsklhl30*	*Loklhl30*	*Frklhl30*	*Saklhl30*	*Omklhl30*	*Drklhl30*	*Caklhl30*	*Cgklhl30-A*
										Caklhl30-A	*Cgklhl30-B*
										Caklhl30-B	
Pmklhl31	*Cmklhl31*	*Lcklhl31*	*Clklhl31*	*Hsklhl31*	*Loklhl31*	*Frklhl31*	*Saklhl31.1*	*Omklhl31.1*	*Drklhl31*	*Caklhl31-A*	*Cgklhl31-A*
							Saklhl31.2	*Omklhl31.2*		*Caklhl31-B*	*Cgklhl31-B*
	Cmklhl32	*Lcklhl32*	*Clklhl32*	*Hsklhl32*	*Loklhl32*	*Frklhl32*	*Saklhl32.1*	*Omklhl32*	*Drklhl32*	*Caklhl32-A*	*Cgklhl32-A*
							Saklhl32.2			*Caklhl32-B*	*Cgklhl32-B*
				Hsklhl33	*Loklhl33*		*Saklhl33.1*	*Omklhl33.1*	*Drklhl33*	*Caklhl33-A*	*Cgklhl33-A*
							Saklhl33.2	*Omklhl33.2*		*Caklhl33-B*	*Cgklhl33-B*
	Cmklhl34	*Lcklhl34*	*Clklhl34*	*Hsklhl34*	*Loklhl34*	*Frklhl34*		*Omklhl34*		*Caklhl34-A*	*Cgklhl34-A*
										Caklhl34-B	*Cgklhl34-B*
		Lcklhl35	*Clklhl35*	*Hsklhl35*	*Loklhl35*		*Saklhl35*	*Omklhl35*	*Drklhl35*	*Caklhl35*	*Cgklhl35*
	Cmklhl36	*Lcklhl36*	*Clklhl36*	*Hsklhl36*	*Loklhl36*	*Frklhl36*	*Saklhl36.1*	*Omklhl36*	*Drklhl36.1*	*Caklhl36-A*	*Cgklhl36-A*
							Saklhl36.2		*Drklhl36.2*	*Caklhl36-B*	*Cgklhl36-B*
Pmklhl37	*Cmklhl37*	*Lcklhl37*	*Clklhl37*	*Hsklhl37*	*Loklhl37*	*Frklhl37a*	*Saklhl37a.1*	*Omklhl37a*	*Drklhl37a*	*Caklhl37a-A*	*Cgklhl37a-A*
							Saklhl37a.2	*Omklhl37a.1*		*Caklhl37a-B*	*Cgklhl37a-B*
							Saklhl37a.3				
						Frklhl37b			*Drklhl37b*	*Caklhl37b-A*	*Cgklhl37b-A*
										Caklhl37b-B	*Cgklhl37b-B*
Pmklhl38	*Cmklhl38*	*Lcklhl38*	*Clklhl38*	*Hsklhl38*	*Loklhl38*	*Frklhl38*	*Saklhl38a.1*	*Omklhl38a.1*	*Drklhl38a.1*	*Caklhl38a-B*	*Cgklhl38a-B*
							Saklhl38a.2	*Omklhl38a.2*	*Drklhl38a.2*		
							Saklhl38a.3				
									Drklhl38b	*Caklhl38b-A*	*Cgklhl38b-A*
Pmklhl39	*Cmklhl39*	*Lcklhl39*	*Clklhl39*	*Hsklhl39*	*Loklhl39*	*Frklhl39*	*Saklhl39a.1*	*Omklhl39a.1*	*Drklhl39a*	*Caklhl39a-A*	*Cgklhl39a-A*
							Saklhl39a.2	*Omklhl39a.2*		*Caklhl39a-B*	*Cgklhl39a-B*
									Drklhl39b	*Caklhl39b-A*	*Cgklhl39b-A*

										Caklhl39b-B	*Cgklhl39b-B1*
											Cgklhl39b-B2
Pmklhl40		*Lcklhl40*	*Clklhl40*	*Hsklhl40*	*Loklhl40*	*Frklhl40a*	*Saklhl40b*	*Omklhl40a*	*Drklhl40a*	*Caklhl40a-A*	*Cgklhl40a-A*
										Caklhl40a-B	*Cgklhl40a-B*
						Frklhl40b			*Drklhl40b*	*Caklhl40b-A*	*Cgklhl40b-A*
	Cmklhl41	*Lcklhl41*	*Clklhl41*	*Hsklhl41*	*Loklhl41*	*Frklhl41a*	*Saklhl41a*	*Omklhl41a.1*	*Drklhl41a*	*Caklhl41a-A*	*Cgklhl41a-A*
								Omklhl41a.2		*Caklhl41a-B*	*Cgklhl41a-B*
						Frklhl41b	*Saklhl41b*	*Omklhl41b.1*	*Drklhl41b*	*Caklhl41b-A*	*Cgklhl41b-A*
								Omklhl41b.2		*Caklhl41b-B*	*Cgklhl41b-B*
	Cmklhl42	*Lcklhl42*	*Clklhl42*	*Hsklhl42*	*Loklhl42*	*Frklhl42*	*Saklhl42.1*	*Omklhl42.1*	*Drklhl42*	*Caklhl42-A*	*Cgklhl42-A*
							Saklhl42.2	*Omklhl42.2*			
					Loklhl43	*Frklhl43*	*Saklhl43.1*	*Omklhl43.1*	*Drklhl43*	*Caklhl43-A*	*Cgklhl43-A*
							Saklhl43.2	*Omklhl43.2*		*Caklhl43-B*	*Cgklhl43-B*

Table S2. Information of 96 *C. auratus klhl* genes.

Name	Position (subject:start:end:strand)	Length of CDS (bp)	Length of amino acid	Isoelectric point	Molecular weight (kDa)
Caklhl2-A	A1:6289232:6285588:-	1803	600	6.1	66.6
Caklhl2-B	B1:15717385:15730870:-	1797	598	6.3	65.4
Caklhl3-A	A14:1978872:1988740:+	1872	623	6.3	73.4
Caklhl4-A	A14:3443901:3465709:+	2133	710	6.4	79.8
Caklhl4-B	B14:20923493:20950021:-	2133	710	8.7	80.4
Caklhl5-A	A1:7241996:7282423:+	2397	798	7.6	86.8
Caklhl5-B	B1:14473532:14517408:-	2289	762	7.1	83.1
Caklhl6-A	A2:5952331:5958088:	1845	614	5.3	69.0
Caklhl6-B	B7:11817521:11822093:+	1845	614	5.6	68.9
Caklhl7-B	B19:3899175:3906635:+	1824	607	6.2	67.9
Caklhl8-1	Contig10482_pilon:35599:42114:	1815	604	5.8	66.8
Caklhl8-2	Contig7794_pilon:68585:74690:-	1815	604	5.8	66.9
Caklhl9-A	A9:15293536:15299379:-	2049	682	5.8	76.0
Caklhl9-B	B9:13882883:13887164:+	2058	685	5.7	76.6
Caklhl10a-B	B17:28581791:28584877:-	1557	518	4.6	58.2
Caklhl10b-A	A17:22045653:22041275:+	1752	583	4.7	65.8
Caklhl10b-B	B17:981585:985705:+	1749	582	4.8	65.9
Caklhl11-A	A12:17643765:17649453:+	2121	706	5.7	80.4
Caklhl11-B	B12:17771742:17854205:+	2211	736	6.0	83.9
Caklhl12-A	A8:16066372:16066979:+	1644	547	5.1	61.3
Caklhl12-B	B8:27763597:27787220:-	1725	574	5.2	64.3
Caklhl13-A	A5:20946030:20982550:+	1923	640	6.3	72.0
Caklhl13-B	B5:7581858:7607171:-	2055	684	6.4	76.9
Caklhl13-like	B4:10805371:10808099:-	1491	496	5.7	56.5
Caklhl14-A	A24:19212275:19222811:-	1833	610	6.0	68.6
Caklhl14-B	B24:18367663:18379021:-	1842	613	6.1	68.8
Caklhl15-A	A24:10381774:10388595:+	1965	654	6.3	75.2
Caklhl15-B	B24:7047580:7061089:	2682	893	5.9	102.7
Caklhl16-B	B18:18055550:18069150:	1827	608	5.7	68.6
Caklhl17a-A	A15:23226276:23230363:+	1983	660	8.7	73.2
Caklhl17a-B	B15:13123159:13128005:+	1989	662	8.6	73.4
Caklhl17b-A	A23:10259946:10268996:+	1773	590	7.1	65.0
Caklhl17b-B	B23:13140305:13150477:+	1776	591	7.1	65.2
Caklhl18-A	A2:9825802:9831246:+	1713	570	5.6	63.5
Caklhl18-B	B2:11292474:11300913:+	1716	571	5.7	63.5
Cakeap1a-A	A2:3385030:3388182:+	1803	600	5.9	67.6
Cakeap1a-B	B2:4348444:4357947:-	1803	600	6.1	67.4
Cakeap1b-A	A6:2152086:2158149:+	1878	625	6.2	70.1
Caklhl20a-A	A3:24898879:24906619:+	1863	620	4.8	69.4

Caklhl20a-B	B3:36207180:36216626:-	1866	621	5.0	69.3
Caklhl20b-A	A2:9489433:9496508:+	1743	580	6.4	65.1
Caklhl20b-B	B2:8646325:8652002:+	1947	648	6.5	72.4
Caklhl21-A	A23:11100645:11103885:	1851	616	5.5	69.6
Caklhl21-B	B23:12240285:12244359:+	1854	617	5.5	69.7
Caklhl22-B	B8:18354485:18365720:-	1887	628	5.5	70.7
Caklhl23.1-A	A9:3527500:3529701:+	1677	558	5.3	63.2
Caklhl23.2-A	A22:18161645:18164148:-	1668	555	5.0	62.7
Caklhl24a-A	A2:1964109:1977292:-	1800	599	6.2	67.9
Caklhl24a-B	B2:2712506:2727522:-	1794	597	6.1	67.7
Caklhl24b-A	B24:13946053:13958275:+	1803	600	6.0	68.2
Caklhl24b-B	A24:15612868:15624288:-	1803	600	6.0	68.1
Caklhl25-A	A25:8289592:8291391:-	1809	602	6.0	66.9
Caklhl25-B	A25:9952839:9954611:+	1800	599	6.3	66.3
Caklhl26-A	A11:5031020:5035356:	1764	587	5.6	66.6
Caklhl26-B	B11:18456046:18462425:+	1818	605	5.7	68.5
Caklhl27-B	B2:26269934:26278675:	1887	628	5.3	70.2
Caklhl28-A	A17:10530670:10532610:	1575	524	5.4	59.1
Caklhl28-B	B17:3905116:3908277:	1575	524	5.5	59.0
Caklhl29a-A	A17:11566226:11680814:+	2433	810	7.1	89.7
Caklhl29a-B	B17:365450:455262:-	2580	859	7.0	94.5
Caklhl29b-A	A20:14807024:14968656:+	2622	873	7.3	94.9
Caklhl29b-B	B20:16205773:16374055:+	2613	870	7.3	94.8
Caklhl30	Contig4209_pilon:661974:665195:	1701	566	5.1	64.3
Caklhl30-A	A6:14682760:14686074:+	1701	566	5.1	64.2
Caklhl30-B	B6:13817342:13820630:-	1689	562	5.0	63.4
Caklhl31-A	A8:11742145:11745424:-	1908	635	6.3	70.9
Caklhl31-B	B13:1719477:1722695:-	1902	633	6.9	70.8
Caklhl32-A	A16:8192688:8206572:+	1839	612	6.2	69.2
Caklhl32-B	B16:18729871:18744134:-	1833	610	6.4	69.1
Caklhl33-A	A7:35742699:35746650:-	2475	824	5.3	93.3
Caklhl33-B	B7:613122:618151:+	2490	829	5.3	93.8
Caklhl34-A	A24:15499551:15501161:+	1677	558	5.2	59.9
Caklhl34-B	B24:13819786:13821543:-	1761	586	5.7	66.2
Caklhl35	Contig13525_pilon:137989:143647:-	1734	577	6.0	65.0
Caklhl36-A	A18:19873705:19876572:+	1914	637	4.9	71.7
Caklhl36-B	B18:18155127:18158245:+	1881	626	5.0	70.3
Caklhl37a-A	A5:22491465:22495298:+	2115	704	6.7	78.0
Caklhl37a-B	B5:11449119:11451325:-	1776	591	6.1	65.9
Caklhl37b-A	A22:15391891:15393663:+	1782	593	6.4	66.0
Caklhl37b-B	B22:26412361:26413971:	1764	587	6.4	59.7
Caklhl38a-B	B19:12879976:12882885:-	1893	630	6.7	66.6

Caklhl38b-A	A16:15768586:15771527:-	1776	591	6.4	67.5
Caklhl39a-A	A20:20838400:20844867:-	2007	668	5.4	74.1
Caklhl39a-B	B20:20671396:20678102:-	2013	670	5.5	74.6
Caklhl39b-A	A2:15413728:15421401:-	1923	640	5.5	71.2
Caklhl39b-B	B2:23147237:23152697:+	1923	640	5.7	71.3
Caklhl40a-A	A2:14491195:14497622:-	1845	614	5.0	69.9
Caklhl40a-B	B2:22198464:22196105:-	1845	614	4.9	69.6
Caklhl40b-A	A24:11929029:11932814:+	1857	618	4.8	70.0
Caklhl41a-A	A9:3493497:3498830:-	1788	595	5.1	67.4
Caklhl41a-B	B9:4915339:4917021:-	1797	598	5.1	67.7
Caklhl41b-A	B6:21752396:21756246:-	1824	607	5.1	68.1
Caklhl41b-B	A6:28618114:28622255:-	1809	602	5.1	68.8
Caklhl42-A	A18:22364629:22367109:+	1698	565	6.7	64.8
Caklhl43-A	A19:11618934:11622036:+	1995	664	7.1	74.1
Caklhl43-B	B19:18216079:18219199:-	1974	657	8.8	73.2

Table S3. Information of 98 *C. gibelio klhl* genes.

Name	Position (subject:start:end:strand)	Length of CDS (bp)	Length of amino acid	Isoelectric point	Molecular weight (kDa)
Cgklhl2-A	A1:2529595:2539200:+	1803	600	6.1	66.6
Cgklhl2-B	B1:12882837:12897653:-	1797	598	6.3	66.6
Cgklhl3-A	A14:1295333:1305547:+	1869	622	6.0	68.9
Cgklhl4-A	A14:2603624:2629418:+	2133	710	6.4	79.7
Cgklhl4-B	B14:26225891:26252571:-	2184	727	8.6	82.4
Cgklhl5-A	A1:3615231:3648836:+	2295	764	7.1	83.3
Cgklhl5-B	B1:11576373:11627782:-	2289	762	7.1	83.1
Cgklhl6-A	A2:14935152:14940909:-	1845	614	5.3	69.0
Cgklhl6-B	B2:5509095:5513680:-	1845	614	5.6	68.9
Cgklhl7-B	B19:31807952:31816106:+	1827	608	6.2	68.0
Cgklhl8-1	scaffold2098:10389:16534:-	1812	603	5.8	66.8
Cgklhl8-2	scaffold1496:26555:53530:-	1815	604	5.8	66.9
Cgklhl8-A	A21:25053782:25059905:+	1815	604	5.8	66.9
Cgklhl8-B	B21:30504088:30510225:-	1815	604	5.8	66.9
Cgklhl9-A	A9:16670634:16677295:-	2064	687	5.9	76.7
Cgklhl9-B	B9:12858189:12862479:+	2064	687	5.7	76.8
Cgklhl10a-B	B17:5861182:5916978:-	1602	533	4.7	60.4
Cgklhl10b-A	A17:27268585:27363537:+	1749	582	4.8	65.9
Cgklhl10b-B	B17:6937727:6942848:+	1752	583	4.7	65.8
Cgklhl11-A	A12:19258102:19263489:+	2112	703	5.7	80.1
Cgklhl11-B	B12:14948764:15045696:-	2211	736	6.0	83.9
Cgklhl12-A	A8:15470484:15486569:-	1728	575	5.0	64.3
Cgklhl12-B	B8:12814684:12838375:+	1728	575	5.1	64.4
Cgklhl13-A	A5:22263163:22289432:-	1983	660	6.2	74.1
Cgklhl13-B	B5:12333405:12358989:+	2049	682	6.4	76.7
Cgklhl13-like	B4:12610939:12613659:-	1491	496	5.7	56.6
Cgklhl14-A	A24:22301317:22323468:+	1878	625	6.1	70.5
Cgklhl14-B	B24:20553383:20564684:+	1821	606	6.1	68.2
Cgklhl15-A	A24:13854993:13858902:-	1836	611	5.8	70.2
Cgklhl15-B	B24:12061832:12075257:-	2682	893	6.0	102.8
Cgklhl16-B	B18:24722729:24728117:+	1824	607	5.7	68.5
Cgklhl17a-A	A15:9247195:9251247:-	1989	662	8.7	73.4
Cgklhl17a-B	B15:21753995:21758840:-	1989	662	8.6	73.4
Cgklhl17b-B1	B23:13055674:13066190:-	1827	608	7.3	65.3
Cgklhl17b-B2	B23:14175222:14185861:+	1779	592	7.1	78.9
Cgklhl18-A	A2:17313037:17318453:-	1719	572	5.6	63.7
Cgklhl18-B	B2:7905926:7914452:-	1749	582	5.7	64.7
Cgkeap1a-A	A2:12423846:12429664:+	1803	600	5.9	67.6
Cgkeap1a-B	B2:2790688:2798743:+	1803	600	6.1	67.4

Cgkeap1b-B	B6:29298866:29305037:-	1881	626	6.2	70.2
Cgklhl20a-A	A3:30876494:30885291:-	1863	620	4.8	65.1
Cgklhl20a-B	B3:44642372:44651950:-	1866	621	5.0	69.0
Cgklhl20b-A	A2:16624415:16630983:-	1743	580	6.4	69.4
Cgklhl20b-B	B2:7215489:7221171:-	1854	617	6.5	69.3
Cgklhl21-A	A23:13895000:13897050:-	1857	618	5.5	70.4
Cgklhl21-B	B23:12146935:12151025:-	1857	618	5.5	70.3
Cgklhl22-B	B8:17882784:17894182:-	1908	635	5.4	71.5
Cgklhl23.1-A	A9:2447749:2450388:-	1668	555	5.0	63.2
Cgklhl23.2-A	A22:21316049:21318553:+	1668	555	5.0	62.7
Cgklhl24a-A	A2:11003685:11017034:-	1800	599	6.2	67.9
Cgklhl24a-B	B2:1384169:1399211:-	1869	622	6.1	70.5
Cgklhl24b-A	A24:18108489:18115758:-	1803	600	6.0	68.2
Cgklhl24b-B	B24:16332199:16344459:-	1803	600	6.0	68.2
Cgklhl25-A	B25:10582525:10584323:-	1761	586	5.9	67.2
Cgklhl25-B	A25:15711961:15713799:+	1803	600	6.2	65.8
Cgklhl26-A	A11:9036655:9040811:+	1773	590	5.6	67.0
Cgklhl26-B	B11:22092326:22099680:-	1818	605	5.7	68.5
Cgklhl27-B	B2:24818504:24827972:-	1767	588	5.2	65.7
Cgklhl28-A	A17:10530670:10532653:+	1575	524	5.4	59.1
Cgklhl28-B	B17:2479060:3593855:+	1575	524	5.5	59.0
Cgklhl29a-A	B17:4384314:4490097:-	2583	860	6.9	94.5
Cgklhl29a-B	B17:5996434:6075082:+	2586	861	7.0	94.5
Cgklhl29b-A	B20:26256832:26415360:-	2622	873	7.3	97.4
Cgklhl29b-B	A20:23982708:24128402:-	2685	894	6.8	94.9
Cgklhl30-A	A6:13363438:13366756:-	1701	566	5.1	64.2
Cgklhl30-B	B6:16651495:16654787:-	1689	562	5.0	63.4
Cgklhl31-A	A13:2214737:2218013:+	1908	635	6.3	70.9
Cgklhl31-B	B13:3849289:3852533:+	1908	635	6.7	71.0
Cgklhl32-A	A16:7319214:7335147:-	1863	620	6.3	70.0
Cgklhl32-B	B16:24055908:24072188:+	1827	608	6.5	68.8
Cgklhl33-A	B15:32963623:32967636:+	2481	826	5.3	93.5
Cgklhl33-B	B7:4023789:4028821:+	2493	830	5.2	93.8
Cgklhl34-A	A24:17987069:17987734:+	1764	587	5.4	65.7
Cgklhl34-B	B24:16205731:16207453:+	1761	586	5.7	66.0
Cgklhl35	scaffold436:796232:802082:+	1707	568	5.8	64.0
Cgklhl36-A	A18:28329722:28333643:+	1914	637	4.9	71.7
Cgklhl36-B	B18:24815267:24819366:+	1902	633	5.0	71.0
Cgklhl37a-A	A5:23785106:23787663:-	1848	615	6.0	68.7
Cgklhl37a-B	B5:10850627:10852831:+	1776	591	6.1	65.9
Cgklhl37b-A	A22:18885248:18887022:-	1773	590	6.2	65.9
Cgklhl37b-B	B22:33137354:33139123:+	1770	589	6.4	65.7
Cgklhl38a-B	B19:11529768:11532679:-	1896	631	6.7	71.9
Cgklhl38b-A	A16:11760786:11763729:-	1791	596	6.5	68.1

Cgklhl39a-A	A20:19193588:19200060:+	2007	668	5.3	74.0
Cgklhl39a-B	B20:21683531:21689935:+	1932	643	5.3	71.4
Cgklhl39b-A	A2:22943653:22951410:-	1923	640	5.6	71.2
Cgklhl39b-B1	B2:13918281:13923724:-	1923	640	5.6	71.2
Cgklhl39b-B2	B2:11214756:11220202:-	1923	640	5.6	71.2
Cgklhl40a-A	A2:22900000:22904834:+	1842	613	5.0	69.8
Cgklhl40a-B	B2:12889825:12892910:-	1845	614	4.9	69.6
Cgklhl40b-A	A24:15630605:15634573:+	1857	618	4.8	70.0
Cgklhl41a-A	A9:2406566:2411446:+	1758	585	5.2	66.2
Cgklhl41a-B	B9:3485229:3489120:+	1773	590	5.1	66.9
Cgklhl41b-A	A6:27677939:27681828:+	1815	604	5.1	68.4
Cgklhl41b-B	B6:31897885:31902007:+	1824	607	5.1	68.9
Cgklhl42-A	A18:30639050:30641530:-	1698	565	6.7	64.8
Cgklhl43-A	A19:13047306:13050402:+	1995	664	7.1	74.1
Cgklhl43-B	B19:17383896:17387024:-	1983	660	9.0	73.7

Table S4. Classification of *Carassius klhl* genes based on chromosome localization and topology.

Category	Gene name	nember
**I**	*klhl2,klhl4, klhl5, klhl6, klhl9, klhl10b, klhl11, klhl12, klhl13,klhl14, klhl15, klhl17a, klhl18, keap1a, klhl20a, klhl20b, klhl21, klhl24a, klhl24b, klhl26, klhl28, klhl29a, klhl29b, klhl31, klhl32, klhl33, klhl34, klhl36, klhl37a, klhl37b, klhl39a, klhl40a, klhl41a, klhl41b, and klhl43*	35
**II**	*klhl30 and klhl39b*	2
**III**	*klhl3, klhl7, klhl10a, klhl13-like, klhl16, keap1b, klhl22, klhl23.1, klhl23.2, klhl27, klhl38a, klhl38b, klhl40b, and klhl42*	14
**VI**	*klhl8, klhl17b, klhl25, and klhl35*	4

Table S5. Identities between *C. gibelio* and *C. auratus* orthologues.

Carassius auratus	***Carsasius gibelio***	**Protein identities**	***Carassius auratus***	***Carsasius gibelio***	**Protein identities**
***Ca*Klhl2-A**	*Cg*Klhl2-A	100.0%	*Ca*Klhl24a-A	*Cg*Klhl24a-A	100.0%
***Ca*Klhl2-B**	*Cg*Klhl2-B	97.9%	*Ca*Klhl24a-B	*Cg*Klhl24a-B	95.9%
***Ca*Klhl3-A**	*Cg*Klhl3-A	91.9%	*Ca*Klhl24b-A	*Cg*Klhl24b-A	100.0%
***Ca*Klhl4-A**	*Cg*Klhl4-A	99.7%	*Ca*Klhl24b-B	*Cg*Klhl24b-B	97.1%
***Ca*Klhl4-B**	*Cg*Klhl4-B	97.2%	*Ca*Klhl25-A	*Cg*Klhl25-A	98.9%
***Ca*Klhl5-A**	*Cg*Klhl5-A	95.3%	*Ca*Klhl25-B	*Cg*Klhl25-B	95.5%
***Ca*Klhl5-B**	*Cg*Klhl5-B	100.0%	*Ca*Klhl26-A	*Cg*Klhl26-A	97.8%
***Ca*Klhl6-A**	*Cg*Klhl6-A	99.6%	*Ca*Klhl26-B	*Cg*Klhl26-B	100.0%
***Ca*Klhl6-B**	*Cg*Klhl6-B	99.8%	*Ca*Klhl27-B	*Cg*Klhl27-B	93.6%
***Ca*Klhl7-B**	*Cg*Klhl7-B	99.8%	*Ca*Klhl28-A	*Cg*Klhl28-A	100.0%
***Ca*Klhl8-1**	*Cg*Klhl8-1	99.5%	*Ca*Klhl28-B	*Cg*Klhl28-B	99.8%
***Ca*Klhl8-1**	*Cg*Klhl8-A	99.6%	*Ca*Klhl29a-A	*Cg*Klhl29a-A	98.8%
***Ca*Klhl8-2**	*Cg*Klhl8-2	99.6%	*Ca*Klhl29a-B	*Cg*Klhl29a-B	86.6%
***Ca*Klhl8-2**	*Cg*Klhl8-B	99.8%	*Ca*Klhl29b-A	*Cg*Klhl29b-A	99.8%
***Ca*Klhl9-A**	*Cg*Klhl9-A	97.9%	*Ca*Klhl29b-B	*Cg*Klhl29b-B	95.2%
***Ca*Klhl9-B**	*Cg*Klhl9-B	99.4%	*Ca*Klhl30	*Cg*Klhl30-A	98.9%
***Ca*Klhl10a-B**	*Cg*Klhl10a-B	68.1%	*Ca*Klhl30-A	*Cg*Klhl30-A	100.0%
***Ca*Klhl10b-A**	*Cg*Klhl10b-A	92.1%	*Ca*Klhl30-B	*Cg*Klhl30-B	100.0%
***Ca*Klhl10b-B**	*Cg*Klhl10b-B	92.1%	*Ca*Klhl31-A	*Cg*Klhl31-A	100.0%
***Ca*Klhl11-A**	*Cg*Klhl11-A	99.2%	*Ca*Klhl31-B	*Cg*Klhl31-B	99.2%
***Ca*Klhl11-B**	*Cg*Klhl11-B	100.0%	*Ca*Klhl32-A	*Cg*Klhl32-A	98.3%
***Ca*Klhl12-A**	*Cg*Klhl12-A	93.2%	*Ca*Klhl32-B	*Cg*Klhl32-B	98.8%
***Ca*Klhl12-B**	*Cg*Klhl12-B	99.2%	*Ca*Klhl33-A	*Cg*Klhl33-A	98.0%
***Ca*Klhl13-A**	*Cg*Klhl13-A	91.3%	*Ca*Klhl33-B	*Cg*Klhl33-B	98.6%
***Ca*Klhl13-B**	*Cg*Klhl13-B	99.5%	*Ca*Klhl34-A	*Cg*Klhl34-A	89.6%
***Ca*Klhl13-like**	*Cg*Klhl13-like	99.1%	*Ca*Klhl34-B	*Cg*Klhl34-B	97.4%
***Ca*Klhl14-A**	*Cg*Klhl14-A	97.2%	*Ca*Klhl35	*Cg*Klhl35	97.7%
***Ca*Klhl14-B**	*Cg*Klhl14-B	98.2%	*Ca*Klhl36-A	*Cg*Klhl36-A	100.0%
***Ca*Klhl15-A**	*Cg*Klhl15-A	91.2%	*Ca*Klhl36-B	*Cg*Klhl36-B	98.4%
***Ca*Klhl15-B**	*Cg*Klhl15-B	99.7%	*Ca*Klhl37a-A	*Cg*Klhl37a-A	85.5%
***Ca*Klhl16-B**	*Cg*Klhl16-B	99.3%	*Ca*Klhl37a-B	*Cg*Klhl37a-B	100.0%
***Ca*Klhl17a-A**	*Cg*Klhl17a-A	99.6%	*Ca*Klhl37b-A	*Cg*Klhl37b-A	97.8%
***Ca*Klhl17a-B**	*Cg*Klhl17a-B	100.0%	*Ca*Klhl37b-B	*Cg*Klhl37b-B	88.7%
***Ca*Klhl17b-A**	*Cg*Klhl17b-B2	82.2%	*Ca*Klhl38a-B	*Cg*Klhl38a-B	91.9%
***Ca*Klhl17b-B**	*Cg*Klhl17b-B1	99.8%	*Ca*Klhl38b-A	*Cg*Klhl38b-A	98.3%
***Ca*Klhl18-A**	*Cg*Klhl18-A	99.6%	*Ca*Klhl39a-A	*Cg*Klhl39a-A	99.7%
***Ca*Klhl18-B**	*Cg*Klhl18-B	97.2%	*Ca*Klhl39a-B	*Cg*Klhl39a-B	94.3%
***Ca*Keap1a-A**	*Cg*Keap1a-A	100.0%	*Ca*Klhl39b-A	*Cg*Klhl39b-A	99.6%
***Ca*Keap1a-B**	*Cg*Keap1a-B	99.8%	*Ca*Klhl39b-B	*Cg*Klhl39b-B1	99.3%

***Ca*Keap1b-A**	*Cg*Keap1b-B	99.5%	*Ca*Klhl39b-B	*Cg*Klhl39b-B2	99.3%
***Ca*Klhl20a-A**	*Cg*Klhl20a-A	99.6%	*Ca*Klhl40a-A	*Cg*Klhl40a-A	99.5%
***Ca*Klhl20a-B**	*Cg*Klhl20a-B	100.0%	*Ca*Klhl40a-B	*Cg*Klhl40a-B	100.0%
***Ca*Klhl20b-A**	*Cg*Klhl20b-A	100.0%	*Ca*Klhl40b-A	*Cg*Klhl40b-A	100.0%
***Ca*Klhl20b-B**	*Cg*Klhl20b-B	94.3%	*Ca*Klhl41a-A	*Cg*Klhl41a-A	98.1%
***Ca*Klhl21-A**	*Cg*Klhl21-A	98.3%	*Ca*Klhl41a-B	*Cg*Klhl41a-B	95.3%
***Ca*Klhl21-B**	*Cg*Klhl21-B	99.0%	*Ca*Klhl41b-A	*Cg*Klhl41b-A	99.3%
***Ca*Klhl22-B**	*Cg*Klhl22-B	98.4%	*Ca*Klhl41b-B	*Cg*Klhl41b-B	99.1%
***Ca*Klhl23.1-A**	*Cg*Klhl23.1-A	100.0%	*Ca*Klhl42-A	*Cg*Klhl42-A	99.5%
***Ca*Klhl23.2-A**	*Cg*Klhl23.2-A	100.0%	*Ca*Klhl43-A	*Cg*Klhl43-A	99.8%
			*Ca*Klhl43-B	*Cg*Klhl43-B	99.6%

Table S6. Identities among alleles of *C. gibelio klhl* genes.

Gene with three alleles	Identities among alleles	Gene with two alleles	Identities between alleles	Gene with one allele
Cgklhl2-A	99.49%-99.77%	*Cgklhl8-1*	99.89%	*Cgklhl18-B*
Cgklhl2-B	99.72%-99.89%	*Cgklhl8-2*	100.00%	
Cgklhl3-A	99.68%-99.73%	*Cgklhl10b-A*	99.32%	
Cgklhl4-A	88.65%-99.59%	*Cgklhl28-B*	99.65%	
Cgklhl4-B	88.12%-95.50%	*Cgklhl29a-A*	99.40%	
Cgklhl5-A	99.61%-99.91%	*Cgklhl39b-B1*	99.53%	
Cgklhl5-B	99.69%-99.84%	*Cgklhl39b-B2*	99.58%	
Cgklhl6-A	99.24%-99.51%			
Cgklhl6-B	99.62%-99.95%			
Cgklhl7-B	99.56%-99.78%			
Cgklhl8-A	99.61%-99.78%			
Cgklhl8-B	99.45%-99.56%			
Cgklhl9-A	99.47%-99.52%			
Cgklhl9-B	99.47%-99.95%			
Cgklhl10a-B	99.06%-99.13%			
Cgklhl10b-B	98.81%-99.08%			
Cgklhl11-A	94.59%-99.76%			
Cgklhl11-B	99.95%-100.00%			
Cgklhl12-A	88.56%-92.27%			
Cgklhl12-B	99.65%-99.82%			
Cgklhl13-A	99.56%-99.85%			
Cgklhl13-B	99.61%-99.76%			
CgKlhl13-like	99.40%-99.80%			
Cgklhl14-A	99.36%-99.63%			
Cgklhl14-B	99.62%-99.73%			
Cgklhl15-A	99.39%-99.54%			
Cgklhl15-B	99.49%-99.95%			
Cgklhl16-B	99.68%-99.84%			
Cgklhl17a-A	99.45%-99.55%			
Cgklhl17a-B	99.95%-100.00%			
Cgklhl17b-B1	99.04%-99.66%			
Cgklhl17b-B2	99.72%-99.83%			
Cgklhl18-A	99.71%-100.00%			
Cgkeap1a-A	99.28%-99.50%			
Cgkeap1a-B	99.56%-99.61%			
Cgkeap1b-B	99.55%-99.94%			
Cgklhl20a-A	99.21%-99.79%			
Cgklhl20a-B	99.46%-99.57%			

Cgklhl20b-A	99.49%-99.66%
Cgklhl20b-B	95.86%-99.90%
Cgklhl21-A	99.24%-99.62%
Cgklhl21-B	99.57%-99.78%
Cgklhl22-B	99.41%-99.63%
Cgklhl23.1-A	99.22%-99.34%
Cgklhl23.2-A	99.46%-99.64%
Cgklhl24a-A	99.28%-99.44%
Cgklhl24a-B	99.39%-99.72%
Cgklhl24b-A	99.47%-99.52%
Cgklhl24b-B	99.79%-99.84%
Cgklhl25-A	99.60%-99.83%
Cgklhl25-B	93.98%-99.79%
Cgklhl26-A	93.91%-98.92%
Cgklhl26-B	99.38%-99.43%
Cgklhl27-B	99.55%-99.58%
Cgklhl28-A	99.24%-99.59%
Cgklhl29a-B	99.23%-99.32%
Cgklhl29b-A	89.13%-99.61%
Cgklhl29b-B	96.94%-99.43%
Cgklhl30-A	99.33%-99.50%
Cgklhl30-B	99.21%-99.49%
Cgklhl31-A	99.84%-99.95%
Cgklhl31-B	99.32%-100.00%
Cgklhl32-A	97.46%-98.93%
Cgklhl32-B	99.41%-99.68%
Cgklhl33-A	98.48%-99.16%
Cgklhl33-B	98.16%-98.63%
Cgklhl34-A	99.66%-99.94%
Cgklhl34-B	98.53%-99.43%
Cgklhl35	99.71%-99.88%
Cgklhl36-A	98.88%-99.39%
Cgklhl36-B	99.58%-99.79%
Cgklhl37a-A	99.44%-99.66%
Cgklhl37a-B	99.44%-99.77%
Cgklhl37b-A	99.15%-99.32%
Cgklhl37b-B	99.49%-99.77%
Cgklhl38a-B	99.54%-99.66%
Cgklhl38b-A	99.27%-99.77%
Cgklhl39a-A	99.69%-99.84%
Cgklhl39a-B	99.64%-99.80%
Cgklhl39b-A	99.38%-99.69%
Cgklhl40a-A	99.29%-99.56%

Cgklhl40a-B	99.67%-99.84%
Cgklhl40b-A	99.08%-99.41%
Cgklhl41a-A	99.22%-99.61%
Cgklhl41a-B	99.28%-99.50%
Cgklhl41b-A	99.01%-99.34%
Cgklhl41b-B	99.34%-99.67%
Cgklhl42-A	98.41%-99.71%
Cgklhl43-A	99.70%-99.85%
Cgklhl43-B	99.70%-99.85%

Table S7. Specific primes for qPCR.

Primer	Sequence	Note
actin-F	AGCACGGTATTGTGACTAACTG	For *C. gibelio* and *C. auratus*
actin-R	TCGAACATGATCTGTGTCATC	
klhl11-A-F	GTGGAGATGAAGGAGTGGAGCAG	
klhl11-A-R	CGCTTCCATTCCACCTGGTCAG	
klhl11-B-F	GCTAACGTGCATGAGGTTCTGGAG	
klhl11-B-R	CGAATCATGTCAGCAGCACGTAAGG	
klhl20a-A-F	GGACGTTGTGAAGGAGTCGATCAG	
klhl20a-A-R	CCTCCTACAGTCACGATCACCTC	
klhl20a-B-F	GTACATCCAGCAGCACTTCAGCAC	
klhl20a-B-R	CCTGATGTGCTCCACAACCTTCTC	
klhl21-A-F	GCTATGATCATACTCTAGACTGCTGG	
klhl21-A-R	GCTGCTACACTGCCTCCAACATG	
klhl21-B-F	CTGGTGGTAGTAGGAGGATGTGATC	
klhl21-B-R	CAGGAGCTGTGGTACTCTCTGG	
Cgkeap1a-A-F	CAAGCAACACTCTCTGGACTGTC	For *C. gibelio*
Cgkeap1a-A-R	GTTGAGAGGTATGACCCAGAGAC	
Cgklhl12-B-F	CTGTCTGGACTTGATGCAGGCAG	
Cgklhl12-B-R	GGTTCGTGCACTCTGCATCTCG	
